# Supplementary material for: Urine tenofovir and dried blood spot tenofovir diphosphate concentrations and viraemia in people taking efavirenz and dolutegravir based antiretroviral therapy
Source: AIDS. Author manuscript; Available in PMC 2024 Apr 1. (PMC7615742; doi:10.1097/QAD.0000000000003818)
Supplement: Figure S1 [file EMS193358-supplement-Figure_S1.docx]

## Figure S1 Receive operating characteristic curves of urine tenofovir and dried blood spot tenofovir diphosphate concentrations to predict viraemia ≥50 copies/mL, by ART regimen


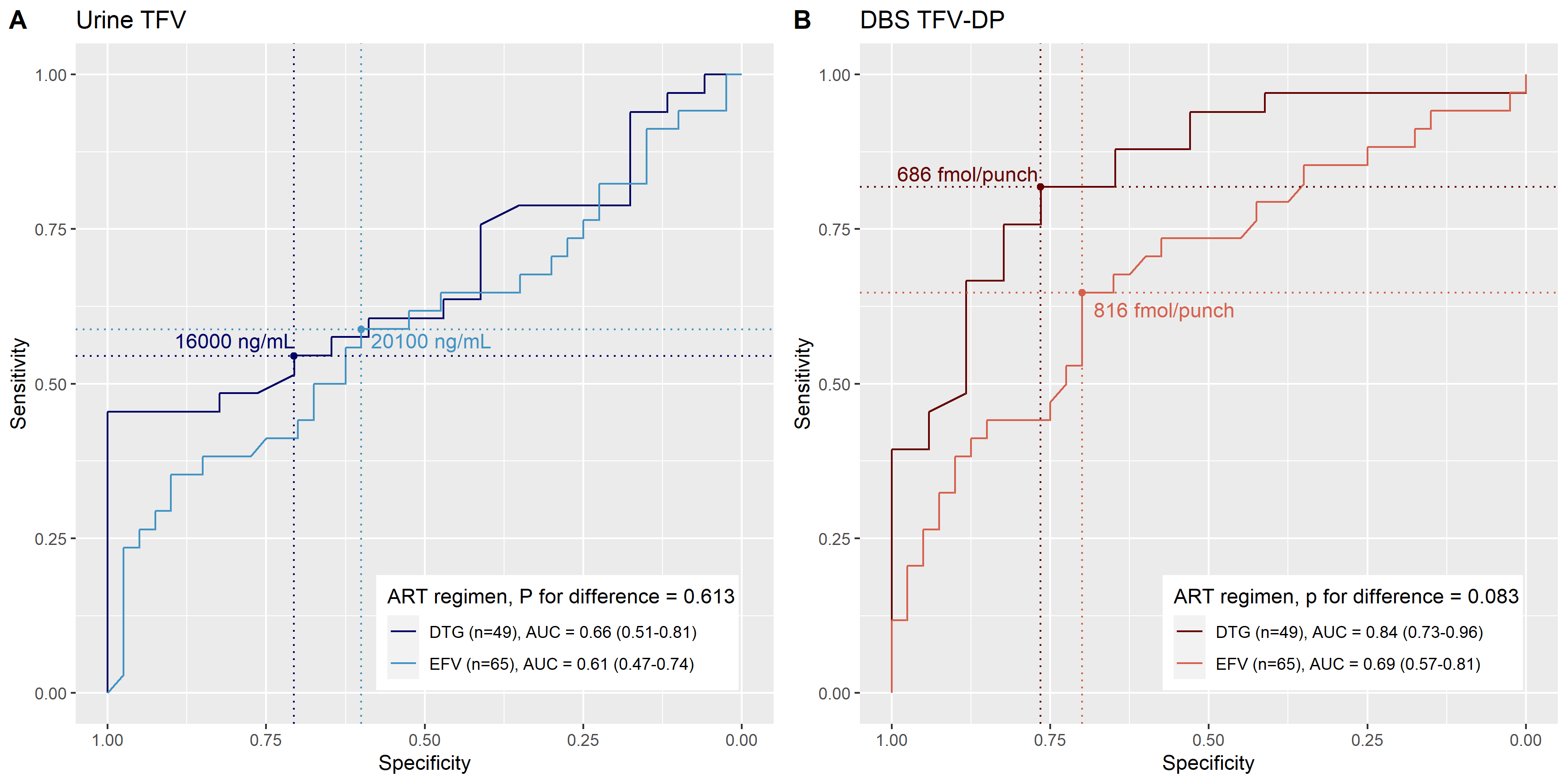


TFV = tenofovir, DBS TFV-DP = dried blood spot tenofovir di-phosphate, ART = antiretroviral therapy, EFV = efavirenz, DTG = dolutegravir, AUC = area under the curve
