# Supplementary material for: Urine tenofovir and dried blood spot tenofovir diphosphate concentrations and viraemia in people taking efavirenz and dolutegravir based antiretroviral therapy
Source: AIDS. Author manuscript; Available in PMC 2024 Apr 1. (PMC7615742; doi:10.1097/QAD.0000000000003818)

## Figure S2 Linear regression models of the association between self-reported adherence measures and urine tenofovir and dried blood spot tenofovir diphosphate concentrations

##
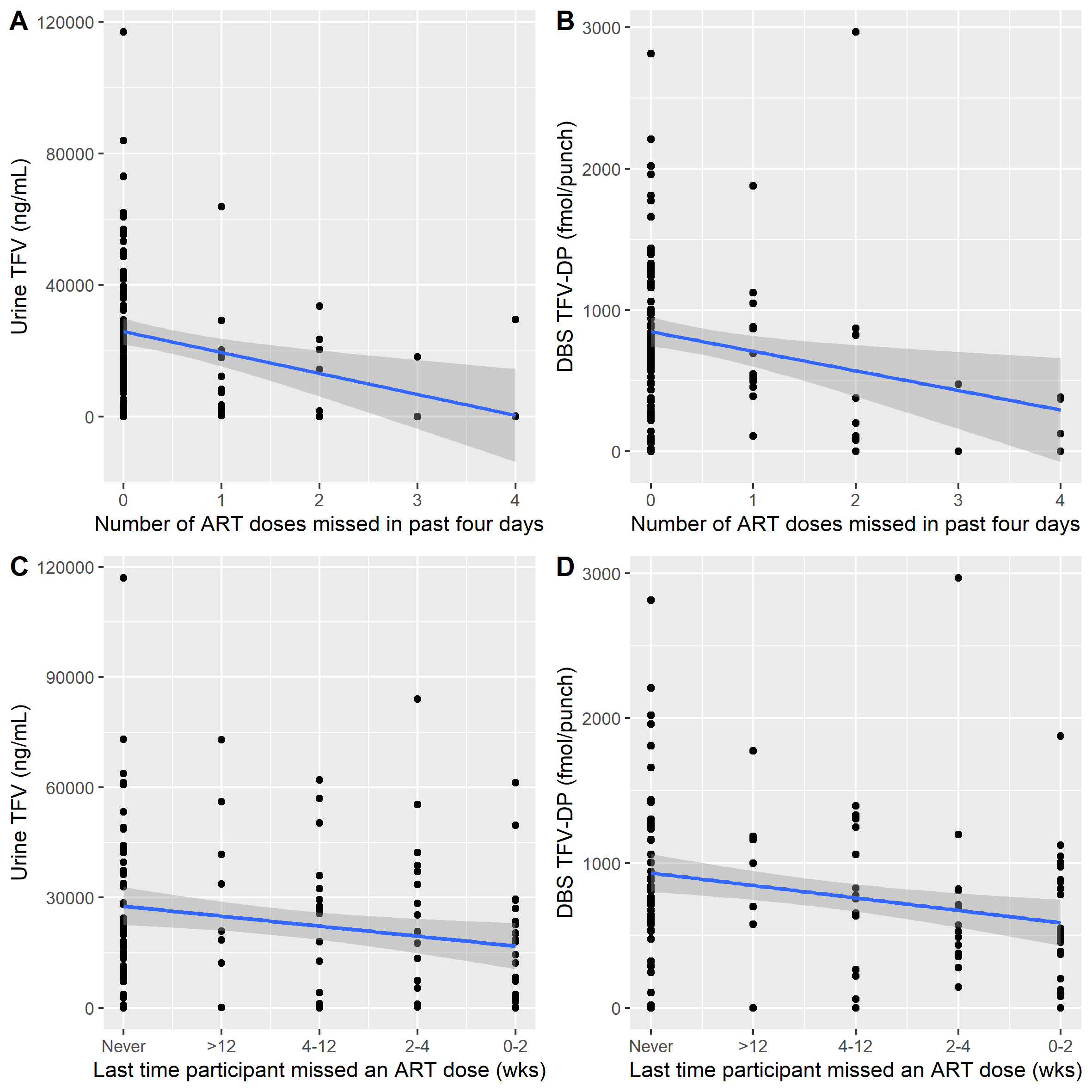

Supplement: Figure S2 [file EMS193358-supplement-Figure_S2.docx]
