# Supplementary material for: Urine tenofovir and dried blood spot tenofovir diphosphate concentrations and viraemia in people taking efavirenz and dolutegravir based antiretroviral therapy
Source: AIDS. Author manuscript; Available in PMC 2024 Apr 1. (PMC7615742; doi:10.1097/QAD.0000000000003818)
Supplement: Table S3 [file EMS193358-supplement-Table_S3.docx]

## Table S3: Median urine TFV and DBS TFV-DP by presence of HIV drug resistance

|  | **No HIV drug resistance** | **HIV drug resistance** |
| --- | --- | --- |
| All (n = 43)* | N = 19 | N = 24 |
| Urine TFV ng/mL, Median (IQR) | 343 (0-20950) | 17300 (1120-29350) |
| DBS TFV-DP fmol/punch, Median (IQR) | 103 (10-374) | 646 (388-820) |
| EFV (n = 28) | N = 4 | N = 24 |
| Urine TFV ng/mL, Median (IQR) | 15490 (3146-35675) | 18100 (1090-29400) |
| DBS TFV-DP fmol/punch, Median (IQR) | 370 (219-634) | 696 (386-828) |
| DTG (n = 14) | N = 15 | N = 1 |
| Urine TFV ng/mL, Median (IQR) | 169 (0-8715) | 3340 (3340-3340) |
| DBS TFV-DP fmol/punch, Median (IQR) | 80 (10-328) | 454 (454-454) |

*Numbers low for meaningful hypothesis testing
