# Supplementary material for: Urine tenofovir and dried blood spot tenofovir diphosphate concentrations and viraemia in people taking efavirenz and dolutegravir based antiretroviral therapy
Source: AIDS. Author manuscript; Available in PMC 2024 Apr 1. (PMC7615742; doi:10.1097/QAD.0000000000003818)
Supplement: Table S4 [file EMS193358-supplement-Table_S4.docx]

## Table S4: Self-reported adherence compared to urine tenofovir and dried blood spot tenofovir diphosphate concentrations

| **Adherence variable** | | **Urine TFV concentration** | | | **DBS TFV-DP** | |
| --- | --- | --- | --- | --- | --- | --- |
|  |  | **Median (IQR) (ng/mL)** | **Coefficient^†^ (95% CI), P, r^2^** | **Median (IQR) (fmol/punch)** | | **Coefficient^†^ (95% CI) , P, r^2^** |
| Number of ART doses missed in past 4 days | 0 | 22600 (11000-37200) | -6380 (-10221 to -2538)  P = 0.001  r^2^ = 0.08 | 783 (572-1161) | | -139 (-238 to -39)  P = 0.007  r^2^ = 0.06 |
|  | 1 | 8125 (3398-18450) |  | 541 (497-879) | |  |
|  | 2 | 1620 (0-20400) |  | 377 (109-824) | |  |
|  | 3 | 9100 (4550-13650) |  | 238 (119-357) | |  |
|  | 4 | 128 (79-7489) |  | 247 (93-374) | |  |
| Last time participant missed a dose of ART (weeks) | Never | 21750 (10800-35675) | -2724 (-4911 to -537)  P = 0.015  r^2^ = 0.05 | 839 (601-1218) | | -86 (-141 to -31)  P = 0.003  r^2^ = 0.07 |
|  | >12 | 27300 (16925-45375) |  | 1080 (668-1183) | |  |
|  | 4-12 | 26300 (3348-33300) |  | 704 (542-1106) | |  |
|  | 2-4 | 23000 (6865-37500) |  | 631 (420-812) | |  |
|  | <2 | 8125 (582-20350) |  | 489 (244-858) | |  |

**^†^**Linear regression models
