# Supplementary material for: Urine tenofovir and dried blood spot tenofovir diphosphate concentrations and viraemia in people taking efavirenz and dolutegravir-based antiretroviral therapy
Source: AIDS. 2024 Jan 8;38(5):697–702. doi: 10.1097/QAD.0000000000003818 (PMC7615742; doi:10.1097/QAD.0000000000003818)
Supplement: Supplementary file 2 [file aids-38-697-s002.docx]

## Figure S1 Receive operating characteristic curves of urine tenofovir and dried blood spot tenofovir diphosphate concentrations to predict viraemia ≥50 copies/mL, by ART regimen

TFV = tenofovir, DBS TFV-DP = dried blood spot tenofovir di-phosphate, ART = antiretroviral therapy, EFV = efavirenz, DTG = dolutegravir, AUC = area under the curve
