# Supplementary material for: Urine tenofovir and dried blood spot tenofovir diphosphate concentrations and viraemia in people taking efavirenz and dolutegravir-based antiretroviral therapy
Source: AIDS. 2024 Jan 8;38(5):697–702. doi: 10.1097/QAD.0000000000003818 (PMC7615742; doi:10.1097/QAD.0000000000003818)
Supplement: Supplementary file 3 [file aids-38-697-s003.docx]

## Figure S2 Linear regression models of the association between self-reported adherence measures and urine tenofovir and dried blood spot tenofovir diphosphate concentrations
