# Supplementary material for: Urine tenofovir and dried blood spot tenofovir diphosphate concentrations and viraemia in people taking efavirenz and dolutegravir-based antiretroviral therapy
Source: AIDS. 2024 Jan 8;38(5):697–702. doi: 10.1097/QAD.0000000000003818 (PMC7615742; doi:10.1097/QAD.0000000000003818)
Supplement: Supplementary file 4 [file aids-38-697-s004.docx]

## Table S1 Baseline characteristics of study population, n= 124

| **Variable** | **Levels** | **Total** |
| --- | --- | --- |
| Age, years | Median (IQR) | 39.0 (34.0 to 45.0) |
| Gender | Female | 68 (54.8) |
|  | Male | 56 (45.2) |
| Ethnicity | Black African | 121 (97.6) |
|  | Coloured | 1 (0.8) |
|  | Other | 2 (1.6) |
| Time since ART initiation, years | Median (IQR) | 4.1 (1.5 to 6.1) |
| Current ART regimen | TDF / FTC / EFV | 74 (59.7) |
|  | TDF / 3TC / DTG | 50 (40.3) |
| Time on current regimen, years | Median (IQR) | 1.6 (0.7 to 5.0) |
| ART side effects | No | 120 (96.8) |
|  | Yes | 4 (3.2) |
| Enrolment CD4 count category, cells/µL | <200 | 21 (16.9) |
|  | 200-349 | 25 (20.2) |
|  | 350-499 | 29 (23.4) |
|  | >=500 | 49 (39.5) |
| Last time participant missed a dose of ART | <2 weeks | 30 (24.2) |
|  | 2-4 weeks | 16 (12.9) |
|  | 1-3 months | 16 (12.9) |
|  | >3 months | 8 (6.5) |
|  | Never | 54 (43.5) |
| Number of ART doses missed in past 4 days | 0 | 95 (76.6) |
|  | 1 | 14 (11.3) |
|  | 2 | 9 (7.3) |
|  | 3 | 2 (1.6) |
|  | 4 | 4 (3.2) |
| Urine tenofovir concentration, ng/mL | Median (IQR) | 20000 (7280 to 33625) |
| Dried blood spot tenofovir diphosphate concentration, fmol/punch | Median (IQR) | 734 (471 to 1015) |
| Enrolment viral load, copies/mL | <50 | 57 (46.0) |
|  | 50-999 | 23 (18.5) |
|  | ≥1000 | 44 (35.5) |
| Any HIV drug resistance against current regimen? | No | 19 (15.3) |
|  | Yes | 24 (19.4) |
|  | Unsuccessful | 1 (0.8) |
|  | Viral load <1000 copies/mL | 80 (64.5) |
