# Supplementary material for: Urine tenofovir and dried blood spot tenofovir diphosphate concentrations and viraemia in people taking efavirenz and dolutegravir-based antiretroviral therapy
Source: AIDS. 2024 Jan 8;38(5):697–702. doi: 10.1097/QAD.0000000000003818 (PMC7615742; doi:10.1097/QAD.0000000000003818)
Supplement: Supplementary file 5 [file aids-38-697-s005.docx]

## Table S2: Sensitivity and specificity of different urine tenofovir and dried blood spot tenofovir diphosphate concentrations to detect viraemia 

| **DTG** |  |  |  |  |  |  |  |  |  |  |  |
| --- | --- | --- | --- | --- | --- | --- | --- | --- | --- | --- | --- |
| **1000 copies/mL** |  |  |  |  |  | **50 copies/mL** |  |  |  |  |  |
| **Urine TFV** |  |  | **DBS TFV-DP** |  |  | **Urine TFV** |  |  | **DBS TFV-DP** |  |  |
| **Urine TFV, ng/mL** | **Sens (%)** | **Spec (%)** | **Urine TFV, ng/mL** | **Sens (%)** | **Spec (%)** | **DBS TFV-DP, fmol/** **punch** | **Sens (%)** | **Spec (%)** | **DBS TFV-DP, fmol/** **punch** | **Sens (%)** | **Spec (%)** |
| Inf | 100.0 | 0.0 | Inf | 100.0 | 0.0 | Inf | 100.0 | 0.0 | Inf | 100.0 | 0.0 |
| 95050 | 100.0 | 3.0 | 1770 | 100.0 | 3.0 | 95050 | 100.0 | 5.9 | 1770 | 97.0 | 0.0 |
| 73000 | 100.0 | 6.1 | 1290 | 100.0 | 6.1 | 73000 | 97.0 | 5.9 | 1290 | 97.0 | 5.9 |
| 67050 | 100.0 | 9.1 | 1224 | 100.0 | 9.1 | 67050 | 97.0 | 11.8 | 1224 | 97.0 | 11.8 |
| 59100 | 94.1 | 9.1 | 1179 | 100.0 | 12.1 | 59100 | 93.9 | 11.8 | 1179 | 97.0 | 17.6 |
| 56150 | 94.1 | 12.1 | 1109 | 100.0 | 15.2 | 56150 | 93.9 | 17.6 | 1109 | 97.0 | 23.5 |
| 52800 | 94.1 | 15.2 | 1029 | 100.0 | 18.2 | 52800 | 90.9 | 17.6 | 1029 | 97.0 | 29.4 |
| 49650 | 94.1 | 18.2 | 969 | 100.0 | 21.2 | 49650 | 87.9 | 17.6 | 969 | 97.0 | 35.3 |
| 46050 | 94.1 | 21.2 | 916 | 100.0 | 24.2 | 46050 | 84.8 | 17.6 | 916 | 97.0 | 41.2 |
| 42700 | 88.2 | 21.2 | 890 | 94.1 | 24.2 | 42700 | 81.8 | 17.6 | 890 | 93.9 | 41.2 |
| 42050 | 88.2 | 24.2 | 879 | 94.1 | 27.3 | 42050 | 78.8 | 17.6 | 879 | 93.9 | 47.1 |
| 40700 | 88.2 | 27.3 | 845 | 94.1 | 30.3 | 40700 | 78.8 | 23.5 | 845 | 93.9 | 52.9 |
| 39150 | 88.2 | 30.3 | 818 | 94.1 | 33.3 | 39150 | 78.8 | 29.4 | 818 | 90.9 | 52.9 |
| 36250 | 88.2 | 33.3 | 813 | 94.1 | 36.4 | 36250 | 78.8 | 35.3 | 813 | 87.9 | 52.9 |
| 33750 | 82.4 | 36.4 | 810 | 94.1 | 39.4 | 33750 | 75.8 | 41.2 | 810 | 87.9 | 58.8 |
| 33350 | 82.4 | 39.4 | 795 | 94.1 | 42.4 | 33350 | 72.7 | 41.2 | 795 | 87.9 | 64.7 |
| 30700 | 82.4 | 42.4 | 768 | 94.1 | 45.5 | 30700 | 69.7 | 41.2 | 768 | 84.8 | 64.7 |
| 27050 | 82.4 | 45.5 | 742 | 94.1 | 48.5 | 27050 | 66.7 | 41.2 | 742 | 81.8 | 64.7 |
| 24400 | 82.4 | 48.5 | 715 | 94.1 | 51.5 | 24400 | 63.6 | 41.2 | 715 | 81.8 | 70.6 |
| 22850 | 82.4 | 51.5 | 686 | 94.1 | 54.5 | 22850 | 63.6 | 47.1 | **686** | **81.8** | **76.5** |
| 21650 | 82.4 | 54.5 | 663 | 94.1 | 57.6 | 21650 | 60.6 | 47.1 | 663 | 78.8 | 76.5 |
| 20650 | 82.4 | 57.6 | 649 | 94.1 | 60.6 | 20650 | 60.6 | 52.9 | 649 | 75.8 | 76.5 |
| 20400 | 82.4 | 60.6 | 617 | 94.1 | 63.6 | 20400 | 60.6 | 58.8 | 617 | 75.8 | 82.4 |
| 19400 | 82.4 | 63.6 | 583 | 94.1 | 66.7 | 19400 | 57.6 | 58.8 | 583 | 72.7 | 82.4 |
| 18100 | 82.4 | 66.7 | 575 | 94.1 | 69.7 | 18100 | 57.6 | 64.7 | 575 | 69.7 | 82.4 |
| 17300 | 82.4 | 69.7 | 560 | 88.2 | 69.7 | 17300 | 54.5 | 64.7 | 560 | 66.7 | 82.4 |
| 16000 | 82.4 | 72.7 | 536 | 88.2 | 72.7 | **16000** | **54.5** | **70.6** | 536 | 66.7 | 88.2 |
| 14250 | 76.5 | 72.7 | 523 | 88.2 | 75.8 | 14250 | 51.5 | 70.6 | 523 | 63.6 | 88.2 |
| 12450 | 76.5 | 78.8 | 509 | 88.2 | 78.8 | 12450 | 48.5 | 76.5 | 509 | 60.6 | 88.2 |
| 10950 | 76.5 | 81.8 | 493 | 88.2 | 81.8 | 10950 | 48.5 | 82.4 | 493 | 57.6 | 88.2 |
| 9740 | 76.5 | 84.8 | **483** | **88.2** | **84.8** | 9740 | 45.5 | 82.4 | 483 | 54.5 | 88.2 |
| 8160 | 76.5 | 87.9 | 465 | 82.4 | 84.8 | 8160 | 45.5 | 88.2 | 465 | 51.5 | 88.2 |
| 5495 | 76.5 | 90.9 | 415 | 76.5 | 84.8 | 5495 | 45.5 | 94.1 | 415 | 48.5 | 88.2 |
| **3495** | **76.5** | **93.9** | 374 | 70.6 | 87.9 | 3495 | 45.5 | 100.0 | 374 | 45.5 | 94.1 |
| 3065 | 70.6 | 93.9 | 362 | 64.7 | 87.9 | 3065 | 42.4 | 100.0 | 362 | 42.4 | 94.1 |
| 2610 | 64.7 | 93.9 | 338 | 64.7 | 90.9 | 2610 | 39.4 | 100.0 | 338 | 39.4 | 94.1 |
| 1521.5 | 58.8 | 93.9 | 304 | 64.7 | 93.9 | 1521.5 | 36.4 | 100.0 | 304 | 39.4 | 100.0 |
| 432.5 | 52.9 | 93.9 | 266 | 58.8 | 93.9 | 432.5 | 33.3 | 100.0 | 266 | 36.4 | 100.0 |
| 244 | 52.9 | 97.0 | 194 | 58.8 | 97.0 | 244 | 30.3 | 100.0 | 194 | 33.3 | 100.0 |
| 221.5 | 52.9 | 100.0 | 125 | 58.8 | 100.0 | 221.5 | 27.3 | 100.0 | 125 | 30.3 | 100.0 |
| 188 | 47.1 | 100.0 | 106 | 52.9 | 100.0 | 188 | 24.2 | 100.0 | 106 | 27.3 | 100.0 |
| 160.5 | 41.2 | 100.0 | 92 | 47.1 | 100.0 | 160.5 | 21.2 | 100.0 | 92 | 24.2 | 100.0 |
| 76 | 35.3 | 100.0 | 80 | 41.2 | 100.0 | 76 | 18.2 | 100.0 | 80 | 21.2 | 100.0 |
| -Inf | 0.0 | 100.0 | 70 | 35.3 | 100.0 | -Inf | 0.0 | 100.0 | 70 | 18.2 | 100.0 |
|  |  |  | 40 | 29.4 | 100.0 |  |  |  | 40 | 15.2 | 100.0 |
|  |  |  | 10 | 23.5 | 100.0 |  |  |  | 10 | 12.1 | 100.0 |
|  |  |  | -Inf | 0.0 | 100.0 |  |  |  | -Inf | 0.0 | 100.0 |
| **EFV** |  |  |  |  |  |  |  |  |  |  |  |
| **1000 copies/mL** |  |  |  |  |  | **50 copies/mL** |  |  |  |  |  |
| **Urine TFV** |  |  | **DBS TFV-DP** |  |  | **Urine TFV** |  |  | **DBS TFV-DP** |  |  |
| **Urine TFV, ng/mL** | **Sens (%)** | **Spec (%)** | **Urine TFV, ng/mL** | **Sens (%)** | **Spec (%)** | **DBS TFV-DP, fmol/** **punch** | **Sens (%)** | **Spec (%)** | **DBS TFV-DP, fmol/** **punch** | **Sens** | **Spec** |
| Inf | 100.0 | 0.0 | Inf | 100.0 | 0.0 | Inf | 100.0 | 0.0 | Inf | 100.0 | 0.0 |
| 73900 | 100.0 | 2.1 | 2892 | 96.3 | 0.0 | 73900 | 100.0 | 2.5 | 2892 | 97.1 | 0.0 |
| 62900 | 96.3 | 2.1 | 2417 | 96.3 | 2.1 | 62900 | 97.1 | 2.5 | 2417 | 97.1 | 2.5 |
| 61650 | 92.6 | 2.1 | 1990 | 96.3 | 4.3 | 61650 | 94.1 | 2.5 | 1990 | 94.1 | 2.5 |
| 61050 | 92.6 | 4.3 | 1919 | 96.3 | 6.4 | 61050 | 94.1 | 5.0 | 1919 | 94.1 | 5.0 |
| 58450 | 92.6 | 6.4 | 1844 | 96.3 | 8.5 | 58450 | 94.1 | 7.5 | 1844 | 94.1 | 7.5 |
| 54700 | 92.6 | 8.5 | 1792 | 96.3 | 10.6 | 54700 | 94.1 | 10.0 | 1792 | 94.1 | 10.0 |
| 51500 | 92.6 | 10.6 | 1716 | 96.3 | 12.8 | 51500 | 91.2 | 10.0 | 1716 | 94.1 | 12.5 |
| 49200 | 92.6 | 12.8 | 1547 | 96.3 | 14.9 | 49200 | 91.2 | 12.5 | 1547 | 94.1 | 15.0 |
| 46400 | 92.6 | 14.9 | 1433 | 92.6 | 14.9 | 46400 | 91.2 | 15.0 | 1433 | 91.2 | 15.0 |
| 43200 | 88.9 | 14.9 | 1425 | 92.6 | 17.0 | 43200 | 88.2 | 15.0 | 1425 | 91.2 | 17.5 |
| 39800 | 85.2 | 14.9 | 1407 | 88.9 | 17.0 | 39800 | 85.3 | 15.0 | 1407 | 88.2 | 17.5 |
| 37200 | 85.2 | 17.0 | 1349 | 88.9 | 19.1 | 37200 | 82.4 | 15.0 | 1349 | 88.2 | 20.0 |
| 36700 | 85.2 | 19.1 | 1303 | 88.9 | 21.3 | 36700 | 82.4 | 17.5 | 1303 | 88.2 | 22.5 |
| 36150 | 85.2 | 21.3 | 1293 | 88.9 | 23.4 | 36150 | 82.4 | 20.0 | 1293 | 88.2 | 25.0 |
| 34800 | 85.2 | 23.4 | 1277 | 88.9 | 25.5 | 34800 | 82.4 | 22.5 | 1277 | 85.3 | 25.0 |
| 33550 | 81.5 | 23.4 | 1257 | 88.9 | 27.7 | 33550 | 79.4 | 22.5 | 1257 | 85.3 | 27.5 |
| 32950 | 77.8 | 23.4 | 1241 | 88.9 | 29.8 | 32950 | 76.5 | 22.5 | 1241 | 85.3 | 30.0 |
| 30950 | 77.8 | 25.5 | 1211 | 88.9 | 31.9 | 30950 | 76.5 | 25.0 | 1211 | 85.3 | 32.5 |
| 29450 | 74.1 | 25.5 | 1183 | 88.9 | 34.0 | 29450 | 73.5 | 25.0 | 1183 | 85.3 | 35.0 |
| 29350 | 74.1 | 27.7 | 1172 | 88.9 | 36.2 | 29350 | 73.5 | 27.5 | 1172 | 82.4 | 35.0 |
| 28900 | 70.4 | 27.7 | 1143 | 88.9 | 40.4 | 28900 | 70.6 | 27.5 | 1143 | 79.4 | 37.5 |
| 28350 | 70.4 | 29.8 | 1092 | 88.9 | 42.6 | 28350 | 70.6 | 30.0 | 1092 | 79.4 | 40.0 |
| 27950 | 66.7 | 29.8 | 1053 | 88.9 | 44.7 | 27950 | 67.6 | 30.0 | 1053 | 79.4 | 42.5 |
| 27350 | 66.7 | 31.9 | 1026 | 85.2 | 44.7 | 27350 | 67.6 | 32.5 | 1026 | 76.5 | 42.5 |
| 26950 | 66.7 | 34.0 | 1003 | 81.5 | 46.8 | 26950 | 67.6 | 35.0 | 1003 | 73.5 | 45.0 |
| 26100 | 63.0 | 34.0 | 987 | 81.5 | 48.9 | 26100 | 64.7 | 35.0 | 987 | 73.5 | 47.5 |
| 24850 | 63.0 | 36.2 | 935 | 81.5 | 51.1 | 24850 | 64.7 | 37.5 | 935 | 73.5 | 50.0 |
| 24150 | 63.0 | 38.3 | 897 | 81.5 | 53.2 | 24150 | 64.7 | 40.0 | 897 | 73.5 | 52.5 |
| 23700 | 63.0 | 40.4 | 889 | 81.5 | 55.3 | 23700 | 64.7 | 42.5 | 889 | 73.5 | 55.0 |
| 23350 | 63.0 | 42.6 | 881 | 81.5 | 57.4 | 23350 | 64.7 | 45.0 | 881 | 73.5 | 57.5 |
| 22950 | 63.0 | 44.7 | 876 | 81.5 | 59.6 | 22950 | 64.7 | 47.5 | 876 | 70.6 | 57.5 |
| 22550 | 59.3 | 44.7 | 858 | 81.5 | 61.7 | 22550 | 61.8 | 47.5 | 858 | 70.6 | 60.0 |
| 22150 | 59.3 | 46.8 | 839 | 77.8 | 63.8 | 22150 | 61.8 | 50.0 | 839 | 67.6 | 62.5 |
| 21750 | 59.3 | 48.9 | 831 | 77.8 | 66.0 | 21750 | 61.8 | 52.5 | 831 | 67.6 | 65.0 |
| 21300 | 59.3 | 51.1 | 826 | 74.1 | 66.0 | 21300 | 58.8 | 52.5 | 826 | 64.7 | 65.0 |
| 20950 | 59.3 | 53.2 | 823 | 74.1 | 68.1 | 20950 | 58.8 | 55.0 | 823 | 64.7 | 67.5 |
| 20650 | 59.3 | 55.3 | **816** | **74.1** | **70.2** | 20650 | 58.8 | 57.5 | **816** | **64.7** | **70.0** |
| **20100** | **59.3** | **57.4** | 794 | 70.4 | 70.2 | **20100** | **58.8** | **60.0** | 794 | 61.8 | 70.0 |
| 19150 | 55.6 | 57.4 | 771 | 66.7 | 70.2 | 19150 | 55.9 | 60.0 | 771 | 58.8 | 70.0 |
| 18350 | 55.6 | 59.6 | 758 | 63.0 | 70.2 | 18350 | 55.9 | 62.5 | 758 | 55.9 | 70.0 |
| 18150 | 51.9 | 59.6 | 744 | 63.0 | 72.3 | 18150 | 52.9 | 62.5 | 744 | 52.9 | 70.0 |
| 18050 | 48.1 | 59.6 | 726 | 63.0 | 74.5 | 18050 | 50.0 | 62.5 | 726 | 52.9 | 72.5 |
| 17800 | 48.1 | 63.8 | 713 | 59.3 | 74.5 | 17800 | 50.0 | 67.5 | 713 | 50.0 | 72.5 |
| 17050 | 48.1 | 66.0 | 704 | 55.6 | 76.6 | 17050 | 47.1 | 67.5 | 704 | 47.1 | 75.0 |
| 15900 | 44.4 | 66.0 | 694 | 51.9 | 76.6 | 15900 | 44.1 | 67.5 | 694 | 44.1 | 75.0 |
| 14850 | 44.4 | 68.1 | 684 | 51.9 | 78.7 | 14850 | 44.1 | 70.0 | 684 | 44.1 | 77.5 |
| 14200 | 40.7 | 68.1 | 663 | 51.9 | 80.9 | 14200 | 41.2 | 70.0 | 663 | 44.1 | 80.0 |
| 13350 | 40.7 | 70.2 | 647 | 51.9 | 83.0 | 13350 | 41.2 | 72.5 | 647 | 44.1 | 82.5 |
| 12450 | 40.7 | 72.3 | 639 | 51.9 | 85.1 | 12450 | 41.2 | 75.0 | 639 | 44.1 | 85.0 |
| 11400 | 40.7 | 76.6 | 626 | 48.1 | 85.1 | 11400 | 38.2 | 77.5 | 626 | 41.2 | 85.0 |
| 10200 | 40.7 | 78.7 | 607 | 48.1 | 87.2 | 10200 | 38.2 | 80.0 | 607 | 41.2 | 87.5 |
| 9105 | 40.7 | 80.9 | 583 | 44.4 | 87.2 | 9105 | 38.2 | 82.5 | 583 | 38.2 | 87.5 |
| 8340 | 40.7 | 83.0 | 552 | 44.4 | 89.4 | 8340 | 38.2 | 85.0 | 552 | 38.2 | 90.0 |
| 8165 | 37.0 | 83.0 | 531 | 40.7 | 89.4 | 8165 | 35.3 | 85.0 | 531 | 35.3 | 90.0 |
| 8020 | 37.0 | 85.1 | 514 | 37.0 | 89.4 | 8020 | 35.3 | 87.5 | 514 | 32.4 | 90.0 |
| 7665 | 37.0 | 87.2 | 491 | 37.0 | 91.5 | 7665 | 35.3 | 90.0 | 491 | 32.4 | 92.5 |
| 7225 | 33.3 | 87.2 | 480 | 37.0 | 93.6 | 7225 | 32.4 | 90.0 | 480 | 29.4 | 92.5 |
| 6255 | 33.3 | 89.4 | 456 | 33.3 | 93.6 | 6255 | 29.4 | 90.0 | 456 | 26.5 | 92.5 |
| 4745 | 33.3 | 91.5 | 412 | 33.3 | 95.7 | 4745 | 29.4 | 92.5 | 412 | 26.5 | 95.0 |
| 3825 | 29.6 | 91.5 | 386 | 29.6 | 95.7 | 3825 | 26.5 | 92.5 | 386 | 23.5 | 95.0 |
| 2830 | 29.6 | 93.6 | 338 | 25.9 | 95.7 | 2830 | 26.5 | 95.0 | 338 | 20.6 | 95.0 |
| 1855 | 29.6 | 95.7 | 285 | 25.9 | 97.9 | 1855 | 23.5 | 95.0 | 285 | 20.6 | 97.5 |
| 1385 | 29.6 | 97.9 | 270 | 22.2 | 97.9 | 1385 | 23.5 | 97.5 | 270 | 17.6 | 97.5 |
| 1090 | 25.9 | 97.9 | 241 | 18.5 | 97.9 | 1090 | 20.6 | 97.5 | 241 | 14.7 | 97.5 |
| 987 | 22.2 | 97.9 | 210 | 14.8 | 97.9 | 987 | 17.6 | 97.5 | 210 | 11.8 | 97.5 |
| 820.5 | 18.5 | 97.9 | 163 | 14.8 | 100.0 | 820.5 | 14.7 | 97.5 | 163 | 11.8 | 100.0 |
| 692 | 14.8 | 97.9 | 117 | 11.1 | 100.0 | 692 | 11.8 | 97.5 | 117 | 8.8 | 100.0 |
| 515 | 11.1 | 97.9 | 55 | 7.4 | 100.0 | 515 | 8.8 | 97.5 | 55 | 5.9 | 100.0 |
| 224 | 7.4 | 97.9 | -Inf | 0.0 | 100.0 | 224 | 5.9 | 97.5 | -Inf | 0.0 | 100.0 |
| 52.5 | 3.7 | 97.9 |  |  |  | 52.5 | 2.9 | 97.5 |  |  |  |
| -Inf | 0.0 | 100.0 |  |  |  | -Inf | 0.0 | 100.0 |  |  |  |
