# Supplementary material for: Urine tenofovir and dried blood spot tenofovir diphosphate concentrations and viraemia in people taking efavirenz and dolutegravir-based antiretroviral therapy
Source: AIDS. 2024 Jan 8;38(5):697–702. doi: 10.1097/QAD.0000000000003818 (PMC7615742; doi:10.1097/QAD.0000000000003818)
Supplement: Supplementary file 7 [file aids-38-697-s007.docx]

## Table S4: Self-reported adherence compared to urine tenofovir and dried blood spot tenofovir diphosphate concentrations

| **Adherence variable** |  | **Urine TFV concentration** |  | **DBS TFV-DP** |  |
| --- | --- | --- | --- | --- | --- |
|  |  | **Median (IQR) (ng/mL)** | **Coefficient**^**†**^ **(95% CI), P, r**^**2**^ | **Median (IQR) (fmol/punch)** | **Coefficient**^**†**^ **(95% CI) , P, r**^**2**^ |
| Number of ART doses missed in past 4 days | 0 | 22600 (11000-37200) | -6380 (-10221 to -2538) P = 0.001 r^2^ = 0.08 | 783 (572-1161) | -139 (-238 to -39) P = 0.007 r^2^ = 0.06 |
|  | 1 | 8125 (3398-18450) |  | 541 (497-879) |  |
|  | 2 | 1620 (0-20400) |  | 377 (109-824) |  |
|  | 3 | 9100 (4550-13650) |  | 238 (119-357) |  |
|  | 4 | 128 (79-7489) |  | 247 (93-374) |  |
| Last time participant missed a dose of ART (weeks) | Never | 21750 (10800-35675) | -2724 (-4911 to -537) P = 0.015 r^2^ = 0.05 | 839 (601-1218) | -86 (-141 to -31) P = 0.003 r^2^ = 0.07 |
|  | >12 | 27300 (16925-45375) |  | 1080 (668-1183) |  |
|  | 4-12 | 26300 (3348-33300) |  | 704 (542-1106) |  |
|  | 2-4 | 23000 (6865-37500) |  | 631 (420-812) |  |
|  | <2 | 8125 (582-20350) |  | 489 (244-858) |  |

^**†**^Linear regression models
